# Information and Communication Technology to Link Criminal Justice Reentrants to HIV Care in the Community

**DOI:** 10.1155/2013/547381

**Published:** 2013-07-28

**Authors:** Ann Kurth, Irene Kuo, James Peterson, Nkiru Azikiwe, Lauri Bazerman, Alice Cates, Curt G. Beckwith

**Affiliations:** ^1^College of Nursing, New York University, New York, NY 10003, USA; ^2^Department of Epidemiology and Biostatistics, The George Washington University School of Public Health and Health Services, Washington, DC 20052, USA; ^3^Department of Medicine, The Miriam Hospital, Alpert Medical School of Brown University, Providence, RI 02912, USA

## Abstract

The United States has the world's highest prison population, and an estimated one in seven HIV-positive persons in the USA passes through a correctional facility annually. Given this, it is critical to develop innovative and effective approaches to support HIV treatment and retention in care among HIV-positive individuals involved in the criminal justice (CJ) system. Information and communication technologies (ICTs), including mobile health (mHealth) interventions, may offer one component of a successful strategy for linkage/retention in care. We describe CARE+ Corrections, a randomized controlled trial (RCT) study now underway in Washington, that will evaluate the combined effect of computerized motivational interview counseling and postrelease short message service (SMS) text message reminders to increase antiretroviral therapy (ART) adherence and linkage and retention in care among HIV-infected persons involved in the criminal justice system. In this report, we describe the development of this ICT/mHealth intervention, outline the study procedures used to evaluate this intervention, and summarize the implications for the mHealth knowledge base.

## 1. Introduction

The criminal justice system in the USA comprised prisons, jails, and community supervision programs including probation and parole and incarcerates more persons than any other nation in the world which disproportionately affects persons of color, the economically disadvantaged, and those who suffer from mental illness [1, 2]. In 2011, there were close to 7 million persons within the CJ system, among which 1.5 million were in prison, 4.5 million were on probation or parole, and close to 750,000 were held in local jails [3]. Prisons typically incarcerate sentenced individuals for periods of one year or greater, whereas jails incarcerate the majority of persons for short periods of time (days, weeks) before releasing them back to the community. A significant proportion of arrests are related to drug and alcohol use [4], with more than 50% of inmates meeting the DSM-IV criteria for drug dependence or abuse [5]. Due to drug laws and punitive sentencing, the criminal justice system is a nexus for large numbers of substance using individuals, many of whom are living with or are at risk for HIV [6]. 

It has been estimated that one in seven HIV-infected persons in the USA passes through correctional facilities in a given year [7], and for these persons, release into the community has been shown to be detrimental to antiretroviral therapy (ART) adherence and maintenance of HIV care [8–10]. New and innovative methods including tools for real-time communication need to be developed to ensure ART adherence and linkage to care for HIV-infected being released from criminal justice facilities. To address these needs, we have developed information-and-communication-technology- (ICT-) based tools to facilitate the delivery of education and counseling regarding the importance of ART and care adherence during the community reentry period. If these tools are found to improve adherence to ART, enhance linkage to community care, and be cost effective, they have the advantage of being able to be readily disseminated throughout the criminal justice system. 

The Washington, DC Department of Corrections (DOC) Central Detention Facility (CDF) has conducted routine opt-out HIV testing since 2006. The HIV prevalence among the Washington, DC DOC population has been estimated to be between 5-6% and among those completing HIV testing upon entrance, close to 1% test positive and among those, 60% represent new HIV diagnoses [11]. These data strongly support the need to apply the Seek, Test, Treat, and Retain strategy [12] to control the HIV epidemic within the DC correctional facilities as part of a broader community strategy to identify persons with HIV, start antiretroviral treatment, and support linkage to and retention in care leading to improved health outcomes and reduced viral load/secondary HIV transmission. To do this effectively, however, new strategies for incarcerated populations are needed [13]. Our study, “CARE+ Corrections”, will assess a combined ICT and mHealth intervention to support HIV linkage to, and retention in, HIV care after jail release in Washington, DC, a city with one of the highest HIV prevalence rates in the USA.

## 2. Materials and Methods

We took a user-centered design [14] approach to adapt two well-developed ICT tools to create the CARE+ Corrections intervention. These tools included the CARE computerized counseling platform (Resources Online, Seattle, WA) and CommCare (Dimagi, Boston MA), an SMS text messaging platform. CARE is a computer-based counseling platform offering HIV risk assessment, tailored counseling, and health promotion planning in versions designed to facilitate rapid HIV testing (Test CARE) and to support ART adherence and secondary HIV prevention (CARE+). The platform uses Microsoft. NET framework with a MySQL backend; a web-based version is now available. The platform was developed using street intercept surveys to review paper prototypes [15], followed by pilot testing and then RCTs. The platform uses narrated self-interviewing to ascertain behavioral risk, assess self-efficacy/motivation, and provide tailored feedback on specific risk behaviors. Prior to developing a health promotion plan around sexual risks or medications, users watch skill-building videos appropriate to their stage of readiness for behavior change. The CARE tool now exists in several forms with different counseling content (rapid HIV testing and primary HIV risk reduction for persons with unknown HIV status and ART adherence and secondary risk reduction for persons already known to be HIV-infected), and it has been adapted for use in several languages (English, Spanish, Kiswahili) and has been used in multiple settings including HIV clinics, community-based organizations, hospitals/emergency departments, and mobile HIV testing services [16–18]. The CommCare SMS platform developed by Dimagi was originally used for data collection by community health workers [19]. In order to make it accessible to all populations, content in the CARE+ tool and the SMS texting intervention is at the 5th grade reading level.

To inform the development of the CARE+ corrections intervention, we conducted formative research with individuals released from either jail or prison (also called “returning citizens”) in the District of Columbia and Rhode Island to determine perceptions of using technology-based tools designed to facilitate linkage to community-based care and viral suppression for HIV-positive jail detainees on ART being released to the community. A complete description of this formative research is reported separately [20], but briefly, 24 qualitative interviews were conducted in Rhode Island (*n* = 12) and Washington DC (*n* = 12) among HIV positive persons with a history of recent incarceration. Participants were asked about their perceptions of the acceptability, usability, and ideas for best practices regarding ICT/mHealth tools including (1) the computer-based counseling intervention; (2) cell phone technology; and (3) text messaging. The returning citizens in this qualitative study reported positive experiences when testing an older version of the CARE+ computerized counseling platform and provided favorable feedback regarding the use of technology-based tools to facilitate linkage to HIV care in the community and adherence to HV medications. Participants with little to no experience using a computer reported feeling comfortable using the tool and felt that the tool would provide more confidentiality than a live counselor. In addition, participants identified additional content that would be relevant for the criminal justice population, including substance use and housing support. 

To develop the SMS text message component of the intervention, we reviewed published and unpublished mHealth resources to develop a library of relevant text messages ([21], W. Curioso personal communication). We then worked with the CommCare team to modify the platform so it could be used to deliver text messages from different subject categories at times and frequencies determined by the participant. In addition, we added flexibility so that participants could alternatively create personalized messages in each subject category. 

## 3. Study Design/Protocol

### 3.1. The CARE+ Corrections Intervention

**Figure 1:**
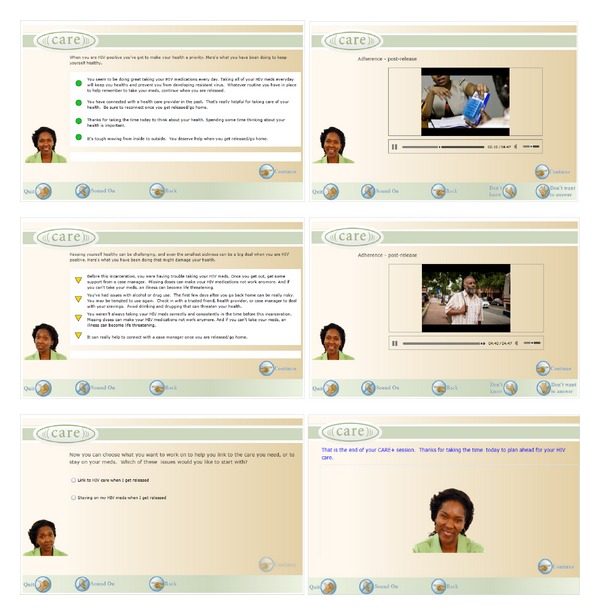
Examples of CARE+ corrections counseling content.

The intervention consists of (1) a counseling session delivered on the CARE platform prior to jail release or soon after release in the community and (2) the SMS text messaging intervention delivered in the community after release. The computerized counseling session on the CARE platform will consist of a one-time, interactive session delivered on a standalone basis on a tablet computer with a touch screen. The session is audio-narrated and 30–40 minutes in length during which participants provide responses to questions about demographic characteristics, sexual risk behaviors and attitudes, substance use, mental health, and HIV treatment and adherence. Based on this assessment, the tool provides tailored feedback messages, displays skill-building videos (topics include barriers to postrelease adherence and linkage to care, partying and HIV, you and your HIV provider, talking about condoms, and tips for remembering your meds) for participants to view to support the delivered feedback, and allows users to make a postrelease health promotion plan to support ART adherence and linkage to community care. Finally, a printout provides a referral list that is customized to meet their identified needs (Figure 1). The counseling session will be delivered 2–4 weeks prior to jail release to HIV-positive detainees recruited inside jail and immediately-after release among persons enrolled through community-based organizations serving this population in the DC area. The goal of the counseling session is to motivate the detainee to anticipate barriers and facilitators to their health care, including linkage to community HIV care and adherence to HIV medications after release.

The cell phone text messaging component of the intervention will be delivered in the community after release from jail. Participants will receive a study cell phone or use an existing personal cell phone for delivery of the text messages to support the participant's linkage plan. Text messages are divided into four distinct categories addressing specific issues related to linkage to care: (1) appointment reminders; (2) medication adherence, (3) HIV secondary prevention, and (4) barriers to care (Table 1). Participants will receive additional administrative text messages related to testing the SMS system and also reminding participants of monthly study calls and regular study visits. A set of prescripted messages will be available for the participant to choose from addressing each of these domains, but participants will also have the opportunity to develop customized messages that may help to encode the messaging to increase confidentiality or that may be more motivating to their needs. For example, instead of the message, “do not forget your upcoming medical appointment. If you cannot make it, call the clinic at xxx-xxx-xxxx,” participants may choose to customize the message to read, “Do not forget your upcoming meeting at the church. If you cannot make it, call the pastor.” For each content category, the participant will be able to choose from several different text-message frequency options (such as daily, every other day, three times weekly, and weekly). The content and frequency of text messages in each content category can subsequently be changed by the participant at the monthly check-in encounters with study staff according to their preference. To maintain confidentiality, text messages will not contain participant names, mention of HIV infection or HIV medications, or specific providers that only provide HIV care. 

The effectiveness of the combined intervention with respect to improving linkage to community HIV care after release and maintaining viral suppression on ART after jail release is being evaluated in a randomized controlled trial among 320 HIV-positive persons in Washington, DC. One-half of the study participants will be randomized to the combined CARE+ Corrections intervention. To achieve a level of intervention equity among study participants, those in the control arm will also view an educational video related to the prevention of overdose following release. All study participants will also receive standard discharge planning services. We will follow all participants for 24 weeks after release for those recruited in the DOC or from the time of study entry for those recruited from the community during which we will conduct follow-up assessments at 12-week and 24-week appointments to determine if linkage to care and adherence to HIV medications were higher in the intervention arm. Monthly check-in phone calls and/or in-person meetings will be used to update locator information and to adjust the content and frequency of text messaging. The main outcome of the trial is the overall proportion of participants in each arm with suppressed HIV viral load; secondary outcomes include attendance at community-based HIV care appointments and self-reported ART adherence. The cost effectiveness of the intervention to support linkage and engagement in care also will be assessed. Using the outcomes as observed within the trial, these analyses will examine the costs per outcome measure from both the correctional system and the community or societal perspective. Because the proposed trial is limited in the duration of observation yet induced benefits or costs may extend beyond the time horizon of the study; we will project future implications of the observed trial outcomes on health and on costs using Markov or simulation models.

The study protocol was approved by the George Washington University IRB (primary), the Miriam Hospital IRB, and the Office of Human Subjects Research Protection (OHRP).

## 4. Conclusions

We will test whether our combined ICT intervention consisting of an interactive tablet-based counseling tool delivered in jail before release or immediately after release combined with an SMS text messaging intervention delivered in the community can support this highly vulnerable group of returning citizens who are living with HIV. These returning citizens often are struggling with active substance use and facing challenges related to poverty, unemployment, and unstable housing all of which create barriers to being retained in continuous HIV care [22]. We hypothesize that this mHealth tool will enable preparatory self-planning and provide ongoing support during community reentry. If found effective and cost effective, we anticipate wide-spread dissemination to criminal justice systems and related community-based organizations that may help address the needs of this vulnerable population and reduce the burden of HIV transmission in the community. 

## Figures and Tables

**Table 1:** CARE+ corrections text message library domains and examples.

Domains	Examples
Library of 10 possible appointment reminder messages or create a customized message	(i) Hey how you feeling? Don't forget to give a call and make your appointment (ii) You're worth it—remember your clinic appointment (iii) Your providers are here to help you—go to your appointment (iv) Call your case manager—he/she can help you get to clinic (v) Don't forget your clinic appointment—it's important (vi) Your health comes first—go to your appointment (vii) Can't remember when your next appointment is? Call the clinic to find out. (viii) Your doctor wants you to come to your appointment (ix) Going to the clinic helps you stay healthy (x) CARE+ Corrections message (staff-inputted from the CARE+ session “plan step groups' subject selected”… Remember, this was your plan: {insert from CARE+ database} (xi) Participant-created message

Medication adherence reminder sent to participant on a selected schedule	(i) Meds keep your body strong and healthy (ii) Don't forget your skittles! (iii) The best way to stay healthy is to take your meds on time and the right way (iv) Adherence to meds means taking your dose at the right time (v) Your meds may not work anymore if you forget to take them (vi) You got to play to win. So don't forget your meds (vii) Call your case manager—he/she can help you find ways to remember to take your meds (viii) Give meaning to your life … Now! (ix) Hey, take your vitamins! (x) CARE+ Corrections message (staff-inputted from the CARE+ session “plan step groups' subject selected”… Remember, this was your plan: {insert from CARE+ database} (xi) Participant-created message

Prevention reminder sent to participant on a selected schedule	(i) Safe sex is important. Use a condom (ii) Don't forget to wrap it or don't give it up! (iii) Did you read “Get your Freak on for Dummies”—it says you must wear a rubber! (iv) Be smart. Use a condom (v) Protect yourself and your partner. Use a condom (vi) If you are using, you may forget your meds (vii) One day at a time. Just for today, don't use (viii) Stay strong. Stay clean (ix) Staying clean is most important. Call your case manager for help (x) CARE+ Corrections message (staff-inputted from the CARE+ session “plan step groups' subject selected”… Remember, this was your plan: {insert from CARE+ database} (xi) Participant-created message

A “barrier to care” reminder message is sent to participant between registration and first check-in appointment (and sent again if person is reincarcerated)	(i) Remember to get a case manager: call xxx-xxx-xxxx (ii) Call your case manager, they're here to help (iii) Hey! Stay linked to your clinic so you can get your meds and care (iv) Need a ride to your appointment? Call your case manager at xxx-xxx-xxxx (v) Can't get your prescriptions? Call your clinic or case manager (vi) Get help for your housing: call xxx-xxx-xxxx (vii) Call transportation services so you can get to your clinic visits: call xxx-xxx-xxxx (viii) Check on job and training programs today (ix) Get help getting your entitlement/insurance programs: call xxx-xxx-xxxx (x) CARE+ Corrections message (staff-inputted from the CARE+ session “plan step groups' subject selected”… Remember, this was your plan: {insert from CARE+ database} (xi) Participant-created message

Welcome message sent during the week after registration	Welcome to the CARE study! We appreciate your participation. Call our staff at xxx-xxx-xxxx if you have any questions

Monthly message reminding participants to schedule their monthly check-in with study staff	Don't forget your monthly check-in meeting. Please call xxx-xxx-xxxx to be sure it is scheduled

## References

[1] Dumont DM, Allen SA, Brockmann BW, Alexander NE, Rich JD (2013). Incarceration, community health, and racial disparities. *Journal of Health Care for the Poor and Underserved*.

[2] International Centre for Prison Studies (ICPS) Entire World-Prison Population Rates per 100,000 of the National Population. http://www.prisonstudies.org/info/worldbrief/.

[3] Glaze LE, Parks E (2012). Bureau of justice statistics (BJS). Correctional populations in the United States, 2011. *NCJ*.

[4] Maston CT (2010). Bureau of Justice Statistics (BJS). Criminal victimization in the United States, 2007, statistical tables. *NCJ*.

[5] James DJ, Glaze LE (2006). Bureau of Justice Statistics (BJS). Mental health problems of prison and jail inmates. *NCJ*.

[6] Centers for Disease Control (CDC) HIV in correctional settings. http://www.cdc.gov/hiv/resources/factsheets/pdf/correctional.pdf.

[7] Spaulding AC, Seals RM, Page MJ, Brzozowski AK, Rhodes W, Hammett TM (2009). HIV/AIDS among inmates of and releasees from US correctional facilities, 2006: declining share of epidemic but persistent public health opportunity. *PLoS ONE*.

[8] Baillargeon J, Giordano TP, Rich JD (2009). Accessing antiretroviral therapy following release from prison. *Journal of the American Medical Association*.

[9] Palepu A, Tyndall MW, Chan K, Wood E, Montaner JSG, Hogg RS (2004). Initiating highly active antiretroviral therapy and continuity of HIV care: the impact of incarceration and prison release on adherence and HIV treatment outcomes. *Antiviral Therapy*.

[10] Stephenson BL, Wohl DA, Golin CE, Tien HC, Stewart P, Kaplan AH (2005). Effect of release from prison and re-incarceration on the viral loads of HIV-infected individuals. *Public Health Reports*.

[11] Beckwith CG, Nunn A, Baucom S (2012). Rapid HIV testing in large urban jails. *American Journal of Public Health*.

[12] Beckwith CG, Zaller ND, Fu JJ, Montague BT, Rich JD (2010). Opportunities to diagnose, treat, and prevent HIV in the criminal justice system. *Journal of Acquired Immune Deficiency Syndromes*.

[13] Thompson MA, Mugavero MJ, Amico KR (2012). Guidelines for improving entry into and retention in care and antiretroviral adherence for persons with HIV: evidence-based recommendations from an International Association of Physicians in AIDS Care panel. *Annals of Internal Medicine*.

[14] McCurdie T, Taneva S, Casselman M (2012). mHealth consumer apps: the case for user-centered design. *Biomedical Instrumentation & Technology*.

[15] Hendry DG, Mackenzie SL, Kurth AE, Spielberg F, Larkin J Evaluating paper prototypes on the street.

[16] Skeels MM, Kurth A, Clausen M, Severynen A, Garcia-Smith H (2006). CARE+ user study: usability and attitudes towards a tablet pc computer counseling tool for HIV+ men and women. *AMIA Annual Symposium Proceedings*.

[17] Spielberg F, Kurth A, Reidy W, McKnight T, Dikobe W, Wilson C (2011). Iterative evaluation in a mobile counseling and testing program to reach people of color at risk for HIV—new strategies improve program acceptability, effectiveness, and evaluation capabilities. *AIDS Education and Prevention*.

[18] Spielberg F, Kurth AE, Severynen A (2011). Computer-facilitated rapid HIV testing in emergency care settings: provider and patient usability and acceptability. *AIDS Education and Prevention*.

[19] Mhila G, DeRenzi B, Mushi C Using mobile applications for community-based social support for chronic patients. http://www.commcarehq.org/pdfs/mhila.pdf.

[20] Peterson J

[21] Dowshen N, Kuhns LM, Johnson A, Holoyda BJ, Garofalo R (2012). Improving adherence to antiretroviral therapy for youth living with HIV/AIDS: a pilot study using personalized, interactive, daily text message reminders. *Journal of Medical Internet Research*.

[22] Springer SA, Spaulding AC, Meyer JP, Altice FL (2011). Public health implications for adequate transitional care for HIV-infected prisoners: five essential components. *Clinical Infectious Diseases*.

